# Prevalence of workplace violence in Northwest Ethiopia: a multivariate analysis

**DOI:** 10.1186/s12912-016-0162-6

**Published:** 2016-07-08

**Authors:** Bewket Tadesse Tiruneh, Berhanu Boru Bifftu, Akililu Azazh Tumebo, Mengistu Mekonnen Kelkay, Degefaye Zelalem Anlay, Berihun Assefa Dachew

**Affiliations:** University of Gondar, College of Medicine and Health Science, School of Nursing, P.O. Box: 196, Gondar, Northwest Ethiopia; Department of Emergency Medicine, School of Medicine, College of Health Sciences, Addis Ababa University, 11706 Addis Ababa, Ethiopia; Department of Epidemiology and Biostatistics, Institute of Public Health, College of Medicine and Health Sciences, University of Gondar, P.O. Box: 196, Gondar, Northwest Ethiopia

**Keywords:** Hospital, Nurse, Prevalence, Factors, Workplace violence

## Abstract

**Background:**

Workplace violence has been acknowledged as a global problem, particularly in the health sector. However, there is scarce data on workplace violence among nurses in Ethiopia. The aim of this study was to assess the prevalence of workplace violence and associated factors among nurses in northwest Ethiopia.

**Methods:**

Hospital based cross-sectional study design was employed in 386 nurses from April 1 – April 30, 2015. Data were collected through the use of self-administered questionnaire developed by the International Labor Office/International Council of Nurses/World Health Organization and Public Services International. To keep the quality of the data collection training was given to supervisors and data collectors. Piloting was done in Debark hospital two weeks before actual data collection to assess the tool’s clarity and make amendments. The proposal was approved by the Institutional Review Board of University of Gondar prior to study commencement and a written consent was obtained from each study participant.

**Results:**

The overall prevalence of workplace violence was 26.7 %. Exploratory logistic regression analyses suggested that age, number of staff in the same work shift, working in a male ward, history of workplace violence, and marital status were factors independently associated with workplace violence

**Conclusion:**

The prevalence of workplace violence among nurses was high. Creating a prevention strategy involving different stakeholders is recommended.

## Background

The definition of workplace violence is: “Incidents where staff are abused, threatened or assaulted in circumstances related to their work, including commuting to and from work, involving an explicit or implicit challenge to their safety, well being or health” [1].

It includes physical and Psychological violence which is a trauma to the victim caused by acts or coercive tactics that includes humiliating the victim, controlling what the victim can and cannot do, withholding information from the victim, deliberately doing something to make the victim feel diminished or embarrassed, isolating the victim from friends and family, and denying the victim access to money or other basic resources [2].

Currently, workplace violence is a widespread problem in the health sector and all health professionals are at a high risk. Gradually, it is becoming a fundamental human rights issue with its negative effect on the achievement of organizations. It affects all sectors and now it is rapidly spreading in the health care setting [3].

In 2013, the Bureau of Labor Statistics (BLS) in the USA reported that workplace assaults ranged from 23,540 and 25,630 annually from which more than 70 % of these were in healthcare and social service settings. Health care and social service workers are almost four times as likely to be injured as a result of violence as the average private sector worker [4].

Violence against healthcare workers is a well-known problem in the United States of America [5]. For instance in Minnesota non-fatal physical assault and non-physical forms of violence are frequent among nurses. Such violence were 13.2 and 38.8 % respectively and mostly they were perpetrated by patients or clients [6].

In addition to physical injury, disability, chronic pain, and muscle tension, nurses who experience violence suffer psychological problems such as loss of sleep, nightmares, and flashbacks as well as short term and long-term emotional reactions, including anger, sadness, frustration, anxiety, irritability, apathy, self-blame, and helplessness. This all can have a direct or indirect relationship with work productivity [6–8]

Today hospital violence against health care providers, especially in nurses, is a major worry in the health care environment, but the actual magnitude of the problem is unknown [9]. In sub-Saharan African countries, nurse reported one or more levels of violence, including physical violence [10]. There is an increasing concern about workplace harassment in South Africa [11].

There is impressive progress of violence in health care service of east African countries; it is tremendously rising among nurses that are highly affected by this vice. Health workers experience different forms of violence both physical and psychological with common forms of violence, including verbal abuse, threats, assault, bullying and harassment. Realities such as staff shortages and increased patient acuity create substantial barriers to eliminating violence [12].

In Ethiopia, researchers showed that nurses had a history of verbal abuse, sexual harassment, bullying/mobbing and physical violence in the past [13]. Therefore, this study was conducted to establish the prevalence and factors associated with workplace violence among nurses working in the referral hospitals of Amhara Regional State, Ethiopia.

## Methods

A hospital based cross-sectional study design was used from April 1 – 30/2015 in three public referral hospitals of Amhara regional state Ethiopia. These were University of Gondar Teaching and Referral Hospital, Felege Hiwot referral hospital, and Deberberhan referral hospital. For each site, the catchment population is estimated to be 7 million people. There were about 800 nurses working in these hospitals. All sampled nurses meeting inclusion criteria and working in the selected study hospitals were included. A single population proportion formula was used to determine the sample size. Assuming a 95 % confidence level, 5 % margin of error and 23.3 % of prevalence [13], the required sample size was at least 292 participating nurses. This sample size was allocated proportionally. Systematic random sampling techniques were used to select participants and participants were asked about their workplace violence experiences for the last 12 months and data were collected using a self-administered questionnaire which was adapted from the International Labor Office/International Council of Nurses/World Health Organization/Public Services International on Workplace Violence in the health sector. The questionnaire was modified in order to meet the local language. To maintain data quality, training was given to data collectors and supervisors. In addition, the questionnaire was pilot tested in 10 % of the total sample size to identify potential problematic areas in Debark Hospital before the actual data collection. Supervision was carried out on daily basis during the data collection to check completeness and consistency of the data both by the supervisor and principal investigator.

### Data analysis

The collected data were entered and cleaned using EPI INFO and analyzed using IBM SPSS Statistics version 20. Frequency distributions and percentages were calculated to describe socio demographic characteristics. Simple and multiple logistic regression analyses were used to explore associations between workplace violence and participant characteristics (working in male ward before, working in male ward now, waiting long time to receive health care, having workplace safety training, availability of enough supplies in the hospital, history of reporting workplace violence,providing community service, having training on anger management, working in the night shift, working any time between 7:00 and 18:00, educational level, sex of the participants, sex of the patient working with, marital states, service year, working in a specific specialties, the number of staff during the same working shift, age of the participants and monthly income). Odds ratios and 95 % confidence intervals were used as measures of association. In an effort to identify characteristics independently associated to workplace violence, an exploratory multivariable logistic model was fitted with the characteristics that were individually associated with workplace violence at the 0.2 significance level. Predictors with *p* < 0.05 in the model were considered suggestive of being independent predictors of workplace violence. No correction for multiple testing was conducted due to the exploratory rather than confirmatory objective of the analysis.

## Results

### Socio-demographic characteristics of the study population

Out of 428 study participants, 386 nurses were participated in the study giving a response rate of 90.2 %. The more than half of the study participants were male 220 [57 %] and 247 [64 %] of them were single. Over three-fourths of the participants were BSc on their educational status [84.5 %]. Nearly half (49.7 %) of the participants have between 1 and 5 years of employment in the hospital (Table 1).

**Table 1:** Socio-demographic characteristics of respondents, Amhara Regional state Referral Hospitals Ethiopia, 2015 (*n* = 386)

Variables		Frequency	%
Age in years	10–29	276	71.5
	30–39	70	18.1
	40–49	25	6.5
	>50	15	3.9
Sex	Male	220	57
	Female	166	43
Marital status	Single	247	64
	Married	21	5.4
	Divorced/Separated	118	30.6
Income/$ USD	<117.5	56	14.5
	117.5–177.5	182	47.2
	178–250	112	29
	>250	36	9.3
Educational level	Diploma	37	9.6
	BSc	328	85
	MSc	21	5.4
Service years	< year	80	20.7
	1–5 years	192	49.7
	6–10	40	10.4
	11–15	29	7.5
	16–20	25	6.5
	>20	20	5.2

### Prevalence of workplace violence among nurses

The number nurses who experienced workplace violence while working in the referral hospitals of the Amhara Regional state was found to be 103 (26.7 %). The prevalence of physical violence was found to be 62 (60.2 %) while 41 (39.8 %) of them were facing the psychological violence. Relatively high (59.2 %) prevalence of work place violence was found among male nurses as compared to females (40.8 %). First degree holder nurses were the most affected group 81 (78.64 %). 38.83 % (40) of the study participants were dissatisfied with the manner how the incident was handled. Among the victims of workplace violence, 36.9 % of them were in need of time off up to one week to recover from the insult.

Those nurses served, 1–5 years were the most frequent victims of workplace violence 41(39.8 %), followed by those nurses whose service year is less than one year 31(30.09). The majority of nurses were attacked by relatives of the patient 62(16.1 %), followed by staff members 28(27.18 %). The largest, 38 (36.9 %) group of the victims experienced the violence between 13:00 and 17:59 h (Table 2).

**Table 2:** Prevalence of Workplace Violence among Nurses Working in the Referral Hospitals of Amhara Regional State, Ethiopia. 2015 (*n* = 386)

Variables		Number (%)
Sex	Male	61(59.2)
	Female	42(40.8)
Age	18–29	71(68.9)
	30–39	16(15.5)
	40–49	11(10.6)
	>50	5(4.85)
Marital status	Single	81(78.6)
	Married	16(15.5)
	Separated	6(5.8)
Educational level	Diploma	17(16.5)
	BSc	81(78.65
	MSc	5(4.85)
Service year	<1 year	31(30.1)
	1–5years	41(39.8)4.9)
	6–10years	5(4.9)
	11–15years	10(9.7)
	16–20years	6(5.8)
	>20 years	10(9.7)
Monthly Income $USD	<117.5	28(27.2)
	117.5–177.5	34(33)
	178–250	30(29.1)
	>250	11(10.67)
Number of staff	1–5 staff	74(71.8)
	6–10 staff	27(26.2)
	>11	2(1.9)
Attackers	Relatives of the patients	62(60.2)
	staff	28(27.18)
	Patient	12(11.65)
	Manager	1(0.97)

Majority 54(52.9 %) of the attackers faced no action from anyone for the unacceptable act while 27 (26.5 %) aggressors experience the consequences, and 22 (21.36 %) participants didn’t know whether measures were taken.

### Factors associated with workplace violence among nurses

In the multivariate logistic regression analysis being age 18–39 (AOR; 0.324, 95 CI: 0.115, 0.919), small number of staff in the same shift (AOR; 2.024, 95 % CI: 1.120, 3.658), working in male ward (AOR; 7.918, 95 % CI: 4.012, 15.626), history of workplace violence (AOR ; 0.270,95 % CI: 0.120,0.610), marital status being single (AOR; 9.153, 95 % CI: 3.593, 13.321) and separated/widowed (AOR; 7.914 95 % CI: 2.036, 9.763) were suggestive of being independent predictors of workplace violence (Table 3).

**Table 3:** Simple and multiple logistic regression analysis of factors associated with Workplace Violence among Nurses working in the Referral Hospitals of Amhara Regional State, Ethiopia. 2015 (*n* = 386)

Variable		WPV		Odds Ratio with 95 % CI		*P* value
		Yes	No	COR	AOR	
Age	18–39	87	259	0.504(0.25,0.99)*	0.324(0.11,0.91)	0.034
	40–55	16	24	1	1	
Number of staff in the same shift	1–5	74	150	2.26(1.38,3.68)*	2.024(1.12,3.65)	0.020
	>5	29	133	1	1	
Working in the night shift	Yes	72	173	1.47(0.910,2.39)	1.788(0.99,3.21)	0.053
	No	31	110	1	1	
Waiting long time to receive care	Yes	54	95	2.18(1.379,3.45)*	1.20(0.61,2.36)	0.57
	No	49	188	1	1	
Work safety training	Yes	39	98	1.16(0.72,1.89)	1.19(0.63,2.25)	0.57
	No	64	185	1	1	
sex	Male	61	159	1.13(0.71,1.79)	1.36(0.68,2.72)	0.38
	Female	42	124	1	1	
Service year	6 m–10 years	87	254	0.62(0.32,1.19)	1.08(0.28,4.06)	0.90
	11–55years	16	29	1	1	
Educational level	BSc	81	245	0.57(0.32,1.02)	1.11(0.48,2.57)	0.80
	Others	22	38	1	1	
Working in male ward	Yes	34	82	1.21(0.74,1.96)	7.92(4.01,15.62)	0.001
	No	69	201	1	1	
History of workplace violence	Yes	13	57	0.57(0.29,1.09)*	0.27(0.12,0.61)	0.002
	No	90	226	1	1	
Marital status	Single	81	166	3.11(1.72,5.63)*	9.15(3.59,13.32)	0.001
	Separated/widowed	6	15	2.55(0.86,7.53)	7.914(2.036,9.763)	0.003
	Married	16	102	1	1	
Working with	Physical disabled	80	169	2.34(1.39,3.95)*	1.64(0.88,3.06)	0.115
	Terminally ill patients	23	144	1	1	

*significantly associated at *P*-value <0.005

## Discussion

Workplace violence is a major problem in the health sector for health professionals. This study showed that the prevalence of workplace violence among nurses working in the Amhara National Regional State was 26.7 %. This is higher than the finding of the previous study, which was done in Oromia National Regional State of Ethiopia [13]. This could be due to differences in the setting of the study.

The prevalence of work place violence in the current study was higher when compared with the findings in USA 25 % [14], Palestine 20.8 % [15], Iran 19 % [16], and Hong Kong 18 % [17]. These may be due to socio economic differences between the countries. This issue can directly expose health care professionals for workplace violence as there is mismatch between health care service need and health care delivery in resource limited countries like Ethiopia. These results in dissatisfaction of health care service consumers in the health care organization so this can lead to workplace violence for health care professionals. Other possible reason can be the difference of the service users in the study areas. In the Amhara National Regional State most of the service users are from the rural community who are not familiarized with the hospital environment, particularly they didn’t know the rooms where to go for each health care services. This will result in unsuccessfulness to get appropriate health care service. This will bring anger from the community side which will definitely exposed nurses for workplace violence more frequently.

In this particular study, the odds of workplace violence among nurses who were single [(AOR; 9.153, 95 % CI: 3.593, 13.321) and separated or widowed (AOR; 7.914 95 % CI: 2.036, 9.763) were nearly 9 and 8 times more likely to have workplace violence as compared to those nurses whose marital status were married respectively. This is in agreement with a study done in Egypt [16].

Moreover, the odds of workplace violence among nurses with 1–5 number of staff during the same working shift were 2 times higher as compared to those nurses who had >11 number of staff during the same working shift(AOR; 2.024, 95 % CI: 1.120, 3.658). This is because when the number of nurses is low in a given shift then patient care and safety could be delayed in the hospital which may results in irritation from the hospital user side so that workplace violence will occur.

Additionally, workplace violence was more likely to be occurred among nurses who were working in the male ward. The odds of workplace violence among nurses who were working in male ward were nearly 8 times higher as compared to nurses who were not work in the male ward (AOR; 7.918, 95 % CI: 4.012, 15.626). This may be due to the fact that males are more aggressive as compared to females. Therefore, those nurses who were assigned in male ward may experience workplace violence more frequently.

Furthermore, the chance of workplace violence among nurses who had history of workplace violence were 88 % less likely to have workplace violence as compared to those nurses who didn’t have history of workplace violence (AOR; 0.270,95 % CI: 0.120,0.610). This may be due fact that, nurses who encountered workplace violence before can develop violence prevention strategy. This is finding is in agreement with a study done in New South Wales [18].

Moreover, nurses who were in the age range of 18–39 were 67.6 % less likely to encountered workplace violence as compared to those nurses who were in the age range of 40–55 (AOR; 0.324, 95 CI: 0.115, 0.919). The reason might be young nurses become effective towards the provision of successful patient care which will definitely decrease the occurrence of workplace violence.

This study has the following limitation. Since the study was cross-sectional, it does not confirm definitive cause and effect relationship. Furthermore, the study may prone to reporting and recall bias.

## Conclusion

In conclusion, this study found that there is high prevalence of workplace violence among nurses working in the referral hospitals of Amhara Regional state. Age, number of staff in the same work shift, working in male ward, history of workplace violence and marital status were the factors independently associated with workplace violence. Creating a prevention strategy involving hospital management body, staff nurses and representatives from nursing association is recommended.

## Abbreviations

BJS, Bureau of Justice and Statistics; BL, Bureau of Labor; NCUS, National Crime Victimization Survey; USA, United States of America; WDR, World Development Report; WHO, World Health Organizations; WPV, workplace violence.
